# Item-Level Investigation of Participant and Study Partner Report on the Cognitive Function Index from the A4 Study Screening Data

**DOI:** 10.14283/jpad.2021.8

**Published:** 2021-03-05

**Authors:** R.E. Amariglio, S.A.M. Sikkes, G.A. Marshall, R.F. Buckley, J.R. Gatchel, K.A. Johnson, D.M. Rentz, M.C. Donohue, R. Raman, C.-K. Sun, R. Yaari, K.C. Holdridge, J.R. Sims, J.D. Grill, P.S. Aisen, R.A. Sperling, A4 Study Team

**Affiliations:** 1 Center for Alzheimer Research and Treatment, Department of Neurology, Brigham and Women's Hospital, Harvard Medical School, 02115, Boston, Massachusetts, USA; 2 Department of Neurology, Massachusetts General Hospital, Harvard Medical School, Boston, Massachusetts, USA; 3 Department of Psychiatry, Massachusetts General Hospital, Harvard Medical School, Boston, Massachusetts, USA; 4 Division of Geriatric Psychiatry, McLean Hospital, Belmont, Massachusetts, USA; 5 Department of Radiology, Massachusetts General Hospital, Harvard Medical School, Boston, Massachusetts, USA; 6 Alzheimer Center Amsterdam, Department of Neurology, Amsterdam University Medical Centers, Amsterdam, Netherlands; 7 Florey Institute, University of Melbourne, Parkville, Victoria, Australia; 8 Melbourne School of Psychological Sciences, University of Melbourne, Parkville, Victoria, Australia; 9 Alzheimer's Therapeutic Research Institute, Keck School of Medicine of the University of Southern California, San Diego, CA, USA; 10 Eli Lilly and Company, Indianapolis, IN, USA; 11 University of California Irvine, Irvine, USA

**Keywords:** Subjective cognitive cecline, amyloid, clinical trial, Alzheimer's disease

## Abstract

**Background:**

Greater subjective cognitive changes on the Cognitive Function Index (CFI) was previously found to be associated with elevated amyloid (Aβ) status in participants screening for the A4 Study, reported by study partners and the participants themselves. While the total score on the CFI related to amyloid for both sources respectively, potential differences in the specific types of cognitive changes reported by either participants or their study partners was not investigated.

**Objectives:**

To determine the specific types of subjective cognitive changes endorsed by participants and their study partners that are associated with amyloid status in individuals screening for an AD prevention trial.

**Design, Setting, Participants:**

Four thousand four hundred and eighty-six cognitively unimpaired (CDR=0; MMSE 25–30) participants (ages 65–85) screening for the A4 Study completed florbetapir (Aβ) Positron Emission Tomography (PET) imaging. Participants were classified as elevated amyloid (Aβ+; n=1323) or non-elevated amyloid (Aβ-; n=3163).

**Measurements:**

Prior to amyloid PET imaging, subjective report of changes in cognitive functioning were measured using the CFI (15 item questionnaire; Yes/Maybe/No response options) and administered separately to both participants and their study partners (i.e., a family member or friend in regular contact with the participant). The impact of demographic factors on CFI report was investigated. For each item of the CFI, the relationship between Aβ and CFI response was investigated using an ordinal mixed effects model for participant and study partner report.

**Results:**

Independent of Aβ status, participants were more likely to report ‘Yes' or ‘Maybe' compared to the study partners for nearly all CFI items. Older age (r= 0.06, p<0.001) and lower education (r=-0.08, p<0.001) of the participant were associated with higher CFI. Highest coincident odds ratios related to Aβ+ for both respondents included items assessing whether ‘a substantial decline in memory' had occurred in the last year (ORsp= 1.35 [95% CI 1.11, 1.63]; ORp= 1.55 [95% CI 1.34, 1.79]) and whether the participant had ‘seen a doctor about memory' (ORsp= 1.56 [95% CI 1.25, 1.95]; ORp =1.71 [95% CI 1.37, 2.12]). For two items, associations were significant for only study partner report; whether the participant ‘Repeats questions' (ORsp = 1.30 [95% CI 1.07, 1.57]) and has ‘trouble following the news' (ORsp= 1.46[95% CI 1.12, 1.91]). One question was significant only for participant report; ‘trouble driving' (ORp= 1.25 [95% CI 1.04, 1.49]).

**Conclusions:**

Elevated Aβ is associated with greater reporting of subjective cognitive changes as measured by the CFI in this cognitively unimpaired population. While participants were more likely than study partners to endorse change on most CFI items, unique CFI items were associated with elevated Aβ for participants and their study partners, supporting the value of both sources of information in clinical trials.

## Introduction

**A**fter a series of disappointing results at the symptomatic stages of Alzheimer's disease (AD), therapeutic trials are increasingly moving towards prevention at the preclinical stage (1). Individuals enrolled in a secondary prevention trial are characterized as clinically normal, but are considered at increased risk of AD due to elevated biomarkers, such as amyloid (Aβ) Positron Emission Tomography (PET) imaging. By shifting the focus earlier in the disease, however, demonstrating clinically meaningful treatment effects is challenging, since most individuals at the preclinical stage are not expected to demonstrate overt cognitive and functional impairment during the course of a trial (2). As such, efforts to identify new methods to capture subtle changes in cognitive functioning prior to the onset of objective clinical impairment are needed to quantify treatment effects with greater resolution.

Subjective report of everyday high-level cognitive functioning from both the individual and a close family member or friend, may offer a window into early cognitive changes along the preclinical stage. Indeed, prior studies have shown that both greater cognitive complaints from the participant, as well as from a study partner, are associated with higher likelihood of subsequent cognitive decline and clinical progression (3, 4, 5, 6).

In addition to serving as outcome measures, subjective cognitive assessments may also facilitate the process of identifying individuals who meet criteria for preclinical AD. As individuals age, cognitive complaints become increasingly common and are not necessarily specific to a pathological process (7). Elucidating particular patterns of complaints from the participant and the study partner that relate to AD biomarkers may enhance the utility of subjective report that is sometimes dismissed for being non-specific (8, 9). Further, better characterization of the subtle changes that are observed at the preclinical stage may ultimately help to identify specific targets for therapeutic intervention.

In the current study, we examined data from individuals screening for the Anti-Amyloid Asymptomatic Alzheimer's (A4) Study testing solanezumab, an anti-amyloid antibody, in a secondary AD prevention trial. In particular, we sought to build upon previous findings in the A4 screen data that found both participant and study partner report related to Aβ on the total score of the Cognitive Function Index (CFI) (10), a subjective questionnaire that asks a participant and study partner about change in the participant's cognitive functioning over the last year (3, 11). Here, we investigated the potential impact of demographic factors on participant vs. study partner report on the total score of the CFI, as well as which specific individual items on the CFI related to amyloid burden on PET. In this way, we aimed to elucidate the pattern of cognitive complaints at the preclinical stage of AD from both the perspective of the participant and study partner.

## Methods

Data presented here come from participants who were screened for the A4 Study. In brief, the A4 Study is a preclinical stage treatment trial that is being conducted at 67 clinical trial sites in the U.S., Canada, Japan, and Australia, among participants with elevated Aβ as determined by florbetapir PET. Participants first underwent an initial clinic screening visit and if eligibility criteria were met, subsequently underwent Aβ PET imaging at a second screening visit. Participants who completed screening for the A4 study, were ages 65–85 years and were considered cognitively unimpaired, based on a global CDR (12) score of 0, Mini-Mental State Exam (MMSE) (13) score of 25–30, and Logical Memory II subscale delayed paragraph recall (LM-IIa) of the Wechsler Memory Scale-Revised (WMS-R) (14) score of 6–18. Moreover, participants did not have unstable or exclusionary medical or psychiatric problems. Participants had adequate literacy in English, Spanish, or Japanese, and had adequate vision and hearing to complete the required cognitive tests. Participants were required to have a study partner who was willing to provide collateral information about the participant's everyday cognitive functioning; study partners were required to have at least weekly contact with participants in person, by phone, or by email. Key exclusion criteria for participants were diagnosis of cognitive impairment or dementia, use of AD medications, unstable anxiety or depression, or other unstable medical conditions, although participants with treated hypertension, diabetes, and other common medical ailments were permitted. Four thousand four hundred and eighty-six participants meeting these criteria then underwent florbetapir PET imaging.

### Cognitive Function Index

The CFI was originally developed as a 14-item, self-administered mail-in screening instrument for AD diagnostic evaluation in prevention trials (10). The CFI has a participant version in which participants report on their own cognitive functioning, as well as a study partner version in which study partners report on the participants' cognitive functioning. The CFI was previously found to have adequate validity and reliability (6, 10). All questions on the CFI ask about cognitive changes over the last year with response options that include Yes (2), No (0), and Maybe (1). Questions range from cognitive items (e.g., “Compared to one year ago, do you feel that your memory has declined substantially?”) to functional items (e.g., “Compared to one year ago, do you have more difficulty managing money?”). The A4 version of the CFI added an additional question, “In the past year, have you seen a doctor about memory concerns?” with response options: Yes (1) or No (0). On a few questions, (e.g., “Has your work performance (paid or volunteer) declined significantly, compared to one year ago?”), Non-Applicable (N/A) was also a response option. A total CFI score can be derived by summing each item of the participant and study partner versions of the questionnaire respectively (the range is 0–29 with higher scores indicating greater complaints about cognitive functioning difficulties). The CFI was administered separately to both the participant and their study partner at the first screen visit prior to florbetapir PET imaging at the second screening visit.

### Amyloid PET Imaging

Florbetapir PET was acquired 50–70 minutes after injection of 10 mCi of florbetapir F 18. Amyloid eligibility (elevated [Aβ+] and eligible to continue in screening vs. not elevated [Aβ−] and ineligible) was assessed using an algorithm combining both quantitative standardized uptake value ratio (SUVr) methodology and qualitative visual read performed at a central laboratory. Mean cortical standardized uptake value ratio [SUVr] using a whole cerebellar reference region of ≥1.15 was utilized to define elevated amyloid as the primary criterion, as quantitative assessment was thought to be more sensitive to the presence of early amyloidosis in the preclinical stage of AD. A SUVr between 1.10 and 1.15 was considered to be elevated amyloid only if the visual read was considered positive by a two independent-reader consensus determination (10).

### Statistical Analyses

Demographic factors of the participant (e.g., sex, age, education) and of the study partner (e.g., partner sex, partner age, living status with participant) were summarized by Aβ status with means, standard deviations, and two-sample t-test; or counts, percentages, and Fisher's Exact test. Pearson's correlation coefficients were calculated for continuous characteristics and CFI scores. Linear regression models were used to determine whether there was an interaction between Aβ status and each participant and study partner characteristic on the CFI score for the participant and study partner report.

To compare level of endorsement on each item of the CFI between participant and study partner, a cumulative odds mixed effects model was employed with CFI response (No=0, Maybe=1, Yes= 2) as the outcome and CFI source (participant or study partner), age, sex, and education as the predictors; and dyad-specific random intercepts. Non-applicable data was treated as missing. Item level missing data was rare and was not analyzed with imputation. To determine the relationship between Aβ status and level of endorsement on each item, separate cumulative odds models were fit for each CFI item. CFI response for participant and study partner were run separately as the dependent variable and Aβ status as the predictor controlling for age, education, and sex. The false discovery rate for item level analysis was adjusted within source (participant vs. study partner) by the method of Benjamini and Hochberg (1995) (15). Statistical analyses were performed using the R software (http://www.r-project.org).

## Results

Of the 4486 participants, 1322 were categorized as Aβ+ (29.5%) and 3163 were Aβ−. Aβ+ participants were slightly older than Aβ− (see table 1). No differences between Aβ groups were observed by sex, years of education, or marital or retirement status. Study partners were majority spouses (62%) and female (60%) and were age 65.8±11.2 years. There were no differences in relationship to study partner by Aβ group.

**Table 1:** Demographic variables of all participants with comparison of Not elevated (Aβ−) and elevated amyloid (Aβ+) groups

Table 1 — Demographics	All Amyloid PET participants N = 4486	Not Elevated Amyloid (Aβ−) N = 3163	Elevated Amyloid(Aβ+) N = 1323	P-value Aβ- vs Aβ+
*Age* — Mean years (S.D.)	71.29 (4.67)	70.95 (4.53)	72.10 (4.89)	<0.0001
*Education* - Mean years (S.D.)	16.58 (2.84)	16.60 (2.85)	16.54 (2.81)	0.5327
*PET SUVr* Mean (S.D.)	1.09 (0.19)	0.99 (0.07)	1.33 (0.18)	< 0.000^1^
*Sex*				0.641^1^
F	2663 (59%)	1885 (60%)	778 (59%)	
*Racial Categories*				
White	4116 (92%)	2866 (91%)	1250 (94%)	< 0.001^1^
Asian	171 (4%)	141 (4%)	30 (2%)	< 0.001^1^
Black or African American	167 (4%)	141 (4%)	30 (2%)	0.007^1^
American Indian or Alaskan Native	32 (1%)	22 (1%)	32 (1%)	0.846^1^
Native Hawaiian	2 (0%)	0 (0%)	2 (0%)	1.00^1^
Unknown or Not Reported	26 (1%)	18 (1%)	8 (1%)	
*Ethnicity*				
Not Hispanic or Latino	4309 (96%)	3040 (96%)	1269 (96%)	0.801^1^
*Marital Status*				0.655^1^
Married	3166 (71%)	2223 (70%)	943 (71%)	
*SP relationship*				0.543
Spouse	2767 (62%)	1948 (62%)	819 (62%)	
Adult child	526 (12%)	236 (13%)	360 (11%)	
Friend/companion	849 (19%)	613 (19%)	236 (18%)	
*Living Situation of SP*				
Living with Participant	3019 (67%)	2131 (67%)	888(67%)	0.889
*SP Gender*				
F	2687 (60%)	1865 (59%)	822 (62%)	0.049^1^
*SP Age* Median years	68.00	68.00	69.00	0.071


1. Fisher's Exact test

Higher score on the participant CFI was associated with older age (r= 0.06, p<0.001) and lower education (r=−0.08, p<0.001), but was not associated with sex (t=−2.68, p=0.79). Study partner CFI score was higher for female study partners (meanfemale= 2.94, meanmale= 2.54; t= 3.5, p=0.0005) and if a study partner lived with the participant (meanlive with= 2.92, meanlive separate= 2.49; t=−3.7, p=0.0003). Older age of the participant related to higher study partner CFI score (r= 0.07, p<0.001).

Next, we were interested in whether demographic factors modified the relationship between Aβ and the CFI total score for participant report and study partner report respectively. When examining participant report, there was not a significant interaction between Aβ and sex (β= −0.14, p=0.30), Aβ and age (β= 0.015, p=0.27), nor Aβ and education level of participant (β= 0.106, p= 0.47) to predict participant CFI score. For study partners, there was not a significant interaction between Aβ and sex of the study partner (β= 0.05, p =0.85) nor Aβ and living situation of study partner with participant (β= 0.17, p=0.53). The interaction between Aβ and age of study partner to predict study partner CFI score was at the significance nominal threshold (β= 0.02, p= 0.05). Specifically, among Aβ−, younger study partners tended to report higher CFI scores. However, among Aβ+, age of study partner did not modify CFI report.

**Figure 1:**
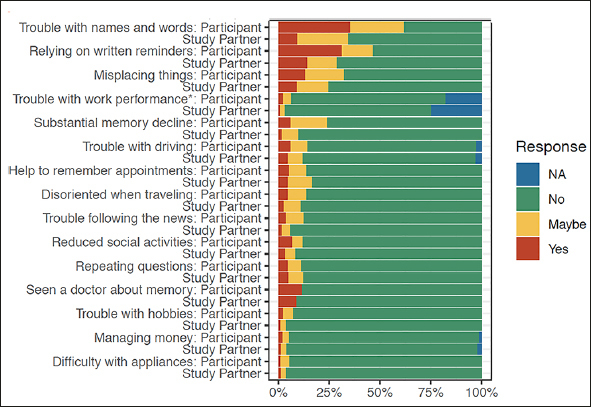
Percentage of endorsement on CFI for participant and study partner report at the item level

At the item level, the 3 most commonly endorsed (‘Yes' or ‘Maybe' response) items were the same for both participant and study partner report (see figure: ‘trouble with names and words' ‘relying on written reminders' and ‘misplacing things' (See figure 1, supplemental table 1). The 3 least endorsed items were also the same for participant and study partner report: ‘managing money' ‘difficulty with hobbies' ‘difficulty with appliances'. For one item, ‘trouble with work performance,' 18% of participants and 25% of study partners did not find this item applicable.

In general, greater endorsement was found for participants compared to their study partners (see figure 1). The only items that did not significantly differ in level of endorsement between participant and study partner included ‘Seen a doctor about memory,' ‘Repeating questions,' ‘Difficulty with appliance and electronic devices.'

**Figure 2:**
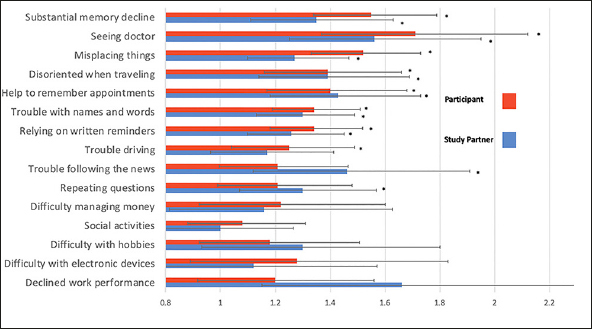
CFI items and odds of endorsement related to Aβ Items presented highest odds of endorsement and Aβ+ to lowest for participant and study partner report. * = significant association of item and Aβ+

As was previously reported (10), Aβ+ participants reported higher total CFI (Cohen d = 0.31, p<0.001) and study partners reported Aβ+ participants had higher total CFI (Cohen d = 0.23, p<0.001). Here, we examined the relationship between Aβ status and level of endorsement for each item. For 7 of the 15 items, Aβ+ was associated with a higher odds of endorsement on CFI for both participant and study partner report and included: ‘Seen a doctor about memory,' ‘Substantial memory decline,' ‘Misplacing things,' ‘Help to remember appointments,' ‘Disoriented when traveling,' ‘Trouble with names and words,' and ‘Relying on written reminders.' For participant report, but not study partner, Aβ+ was associated with greater endorsement on ‘trouble with driving.' For study partner report but not participant, Aβ+ was associated with ‘trouble following the news' and ‘repeating questions' (see figure 2). Additionally, Aβ+ was associated with ‘decline in work performance' but as noted above 25% of study partners answered ‘not applicable' leading to less stable estimates on this item compared to other items on the CFI. All items significantly associated with Aβ+ remained significant after an FDR adjustment.

## Discussion

Extending previous findings from the A4 screen data that found for Aβ+ participants, participant and study partner CFI scores were higher than for Aβ- participants (10), here we found at an item-level, 7 of the 15 CFI items were related to Aβ+ for participant and study partner report. Further, the odds of endorsement associated with elevated Aβ was numerically higher for participant report compared to study partner report. Items that were related to both study partner and participant report reflected predominantly cognitive changes (e.g., ‘Substantial memory decline,' ‘Misplacing things'). Functional items (e.g., ‘Hobbies more difficult,' ‘Reduced social activities,' ‘Difficulty managing money,' and ‘Difficulty with appliances') were much less likely to be endorsed by either a participant or a study partner and were not associated with Aβ+.

Additionally, the item ‘seen a doctor about memory' related to Aβ+ for both participant and study partner report. Unlike the other CFI items, this question is based on a specific event rather than an overall subjective experience. The association of ‘seen a doctor about memory' with Aβ is consistent with previous work demonstrating individuals recruited from a memory clinic are more likely Aβ+ compared to community-based individuals with subjective memory complaints (9, 16).

Several items were only related to study partner or participant report alone. Specifically, two items were related to Aβ+ for study partner report (i.e., ‘Repeating questions' and ‘trouble following the news'). Interestingly, the fact that study partners report ‘repeating questions,' but not participants themselves is consistent with clinical observations, in that the participant may not realize they are repeating themselves and would be less likely to endorse this item.

These findings suggest that, even at the preclinical phase, subtle changes in cognitive functioning are recognized by and at the level of awareness of some participants and some study partners. Participant report, while potentially sensitive at the earliest stages of disease, has faced criticism as an outcome in clinical trials as changes in self-awareness (i.e., anosognosia) in patient reported outcomes can occur, particularly by the stage of dementia (17). Thus, there have been concerns as to whether participant report can reliably serve as an indicator of symptom progression for the duration of a trial as individuals move towards clinical impairment. Conversely, it has been unclear what changes if any, a study partner can observe at the preclinical stage when individuals are entirely independent in their daily activities. Importantly, in this study of individuals screening for a prevention trial, study partner report was consistent with participant report, despite the fact that individuals had a global CDR score of 0.

We also examined the impact of demographic features of participants and their study partners on the relationship between Aβ status and CFI total score. For participants, while there was a higher level of endorsement for CFI that related to older age and lower education, these demographic variables did not significantly modify the relationship between Aβ status and CFI score. Likewise, there was a higher level of endorsement for study partner-reported CFI if the study partner was female or lived with the participant, however these demographic variables did not significantly modify the relationship between Aβ status and CFI score. Finally, while age of the study partner did not relate to study partner reported CFI score, there was a nominally significant modifying effect of study partner age on the relationship between Aβ status and CFI score. Taken together, the associations between Aβ+ and CFI seem to remain even after after adjusting for age, education, sex, and living situation of study partner with participant.

A few limitations to this study a worth noting. Participants in this study were highly educated with limited ethnic and racial diversity, typical for clinical trial populations. Thus, it remains unknown how these findings might generalize to the larger population, as there may be educational and cultural differences in the value of subjective reporting as it relates to risk for AD.

Our findings are in support of subjective report of cognitive functioning to characterize early manifestations of AD among those in an early-treatment trial. While participant report appears to be numerically higher as it relates to Aβ status compared study partner report, study partners are also observing similar changes as they relate to Aβ status. In the future, examining items longitudinally with tau PET in combination with amyloid PET will also help to better approximate the optimal utility of participant and study partner report as individuals decline or improve during the course of a trial.

## Electronic supplementary material

Supplementary material, approximately 88.4 KB.

*Funding:* The A4 Study is a secondary prevention trial in preclinical Alzheimer's disease, aiming to slow cognitive decline associated with brain amyloid accumulation in clinically normal older individuals. The A4 Study is funded by a public-private-philanthropic partnership, including funding from the National Institutes of Health-National Institute on Aging (U19 AG010483, U24AG057437, R01 AG063689), Eli Lilly and Company, Alzheimer's Association, Accelerating Medicines Partnership, GHR Foundation, an anonymous foundation and additional private donors, with in-kind support from Avid and Cogstate. The companion observational Longitudinal Evaluation of Amyloid Risk and Neurodegeneration (LEARN) Study is funded by the Alzheimer's Association (LEARN-15-338729) and GHR Foundation. The A4 and LEARN Studies are led by Dr. Reisa Sperling at Brigham and Women's Hospital, Harvard Medical School and Dr. Paul Aisen at the Alzheimer's Therapeutic Research Institute (ATRI), University of Southern California. The A4 and LEARN Studies are coordinated by ATRI at the University of Southern California, and the data are made available through the Laboratory for Neuro Imaging at the University of Southern California. The participants screening for the A4 Study provided permission to share their de-identified data in order to advance the quest to find a successful treatment for Alzheimer's disease.

*Conflict of Interest:* R. Amariglio has nothing to disclose. J. Grill has nothing to disclose. D. Rentz has nothing to disclose. G Marshall reports personal fees and institutional support from Eisai Inc., institutional support from Eli Lilly and Company, Janssen Alzheimer Immunotherapy, Novartis, and Genentech, and personal fees from Grifols Shared Services North America, Inc, Pfizer outside the submitted work. R. Buckley has nothing to disclose. R. Yaari reports personal fees from Eli Lilly and Company during the conduct of the study. R. Raman reports grants from NIA, grants from Eli Lilly during the conduct of the study, grants from Janssen and grants from Eisai, outside the submitted work. CK. Sun reports grants from NIA and grants from Eli Lilly and Company during the conduct of the study. J. Sims he is an employee and stock holder of Eli Lilly and Company outside the submitted work. M. Donohue reports grants from NIH, grants and personal fees from Eli Lilly and Company during the conduct of the study, personal fees from Roche, personal fees from Biogen, personal fees from Neurotrack, other from Janssen, personal fees from Vivid Genomics outside the submitted work. P. Aisen reports grants from Janssen, grants from NIA, grants from FNIH, grants from Alzheimer's Association, grants from Eisai, personal fees from Merck, personal fees from Biogen, personal fees from Roche, personal fees from Lundbeck, personal fees from Proclara, personal fees from Immunobrain Checkpoint outside the submitted work. K. Holdrige reports she is an employee and minor stockholder of Eli Lilly and Company. S Sikkes reports grants from Zon-MW OffRoad, grants from EU-JPND, institutional support from Lundbeck, Boehringer, and Toyama outside the submitted work. Dr. Gatchel reports grants from Alzheimer's Association, grants from NIH/NIA, personal fees from Huron, grants from Merck, outside the submitted work.

*Ethical Standards:* Study protocols were approved by the Partners Institutional Review Board, and all participants provided written informed consent before undergoing any study procedures.”
