# Supplementary material for: Item-Level Investigation of Participant and Study Partner Report on the Cognitive Function Index from the A4 Study Screening Data
Source: J Prev Alzheimers Dis. Author manuscript; Available in PMC 2021 Jun 29. (PMC8240963; doi:10.14283/jpad.2021.8)
Supplement: Supplementary file 1 [file NIHMS1706889-supplement-Supplemental_Table_1.docx]

Supplemental Table 1. Percentage of endorsement for participant and study report on each item of the CFI

|  | **PARTICIPANT** |  |  |  | **STUDY PARTNER** |  |  |  |
| --- | --- | --- | --- | --- | --- | --- | --- | --- |
|  | YES | MAYBE | NO | N/A | YES | MAYBE | NO | N/A |
| **Trouble with names and words** | 34.39 | 26.07 | 37.48 | --- | 9.9 | 27.34 | 72.36 | --- |
| **Relying on written reminders** | 30.99 | 15.25 | 53.57 | --- | 13.92 | 14.67 | 71.22 | --- |
| **Misplacing things** | 12.93 | 19.08 | 67.78 | --- | 8.89 | 15.42 | 75.48 | --- |
| **Trouble with work performance*** | 2.05 | 3.92 | 76.0 | 17.99 | 0.63 | 2.26 | 72.22 | 24.85 |
| **Substantial memory decline** | 5.89 | 17.81 | 76.29 | --- | 1.43 | 7.98 | 87.97 | --- |
| **Trouble with driving** | 5.79 | 8.05 | 83.35 | 2.87 | 4.38 | 7.02 | 84.37 | 3.16 |
| **Help to remember appointments** | 4.92 | 8.38 | 86.0 | --- | 4.79 | 12.37 | 87.67 | --- |
| **Disoriented when traveling** | 4.34 | 9.1 | 86.33 | --- | 2.47 | 8.08 | 88.95 | --- |
| **Trouble following the news** | 3.43 | 8.63 | 87.83 | --- | 1.36 | 4.23 | 94.3 | --- |
| **Reduced social activities** | 6.51 | 5.22 | 88.25 | --- | 2.96 | 5.24 | 91.78 |  |
| **Repeating questions** | 4.35 | 6.57 | 89.0 | --- | 4.64 | 7.33 | 87.95 | --- |
| **Seen a doctor about memory** | 11.71 | --- | 90.68 | --- | 8.52 | --- | 90.48 | --- |
| **Trouble with hobbies** | 2.05 | 4.88 | 92.81 | --- | 0.73 | 2.9 | 96.11 | --- |
| **Managing money** | 1.8 | 3.17 | 93.66 | 1.27 | 1.02 | 2.7 | 93.84 | 2.34 |
| **Difficulty with appliances** | 0.77 | 4.42 | 94.86 | --- | 0.99 | 2.5 | 96.37 | --- |

CFI=Cognitive Function Index
